# Factors associated with caregiving burden and mental health conditions in caregivers of patients with anorexia nervosa in Japan

**DOI:** 10.1186/s13030-016-0073-5

**Published:** 2016-06-22

**Authors:** Chisato Ohara, Gen Komaki, Zentaro Yamagata, Mari Hotta, Toshiko Kamo, Tetuya Ando

**Affiliations:** Institute of Women’s Health, Tokyo Women’s Medical University 9-9, Wakamatu-cyo, Shinzyuku-ku, Tokyo, 162-0056 Japan; Department of Psychosomatic Research, National Institute of Mental Health, National Center of Neurology and Psychiatry, 4-1-1, Ogawahigashi-cyo, Kodaira-shi, Tokyo, 187-8553 Japan; School of Health Sciences Fukuoka, International University of Health and Welfare, 137-1 Enokizu, Ohkawa, Fukuoka, 831-8501 Japan; Department of Health Sciences, School of Medicine, University of Yamanashi, 1110, Shimokato, Chuo, Yamanashi 409-3898 Japan; Health Services Center, National Graduate Institute for Policy Studies, 7-22-1, Roppongi, Minato-ku, Tokyo, 106-8677 Japan

## Abstract

**Background:**

There are no studies about the caregiving burdens in families of patients with eating disorders in Japan, and only limited studies on the role of caregivers’ stress coping, social support, and mental health. This study examines caregiving burdens, mental health conditions, and associated factors in caregivers of anorexia nervosa (AN) patients in Japan.

**Methods:**

Seventy-nine principal caregivers (70 mothers, 5 fathers, 3 spouses and 1 grandmother; mean age 56.0 ± 8.0 years) for outpatients with AN (all female; mean age 26.6 ± 7.9 years; BMI 14.6 ± 3.2 kg/m^2^) were evaluated using self-report questionnaires in a cross-sectional study. The questionnaires included caregiving burden (J-ZBI_8), mental health conditions (GHQ28), stress coping styles (CISS), social support (SNQ), severity of the patient’s symptoms from the family’s perspective (ABOS), and family functioning (GF-FAD). Clinical information about the patients was also obtained.

**Results:**

Mean caregiving burden assessed by J-ZBI_8 score was 12.4 ± 7.0 (SD). The total GHQ score was 31.6 ± 13.7 (Likert scoring) and 9.2 ± 7.0 (GHQ scoring). Of the respondents, 48 (60.7 %) indicated a high risk for mental health problems that exceeded the cutoff point of the GHQ. Significantly higher caregiving burden and poor mental health conditions were shown in the group who had contact with patients > 6 h a day compared to the group with daily patient contact < 3 h (F (2, 76) = 3.19, *p* = 0.047 and F (2, 76) = 9.39, *p* < 0.001, respectively). Stepwise multiple regression analysis indicated that the factors that significantly predicted the caregiving burden were severity of the patient’s symptoms from the family’s perspective (*β* = 0.47, *p* < 0.001) and Emotion-Oriented Coping (*β* = 0.38, *p* = 0.002) (R^2^ = 0.401), while predictors of mental health conditions were Emotion-Oriented Coping (*β* = 0.522, *p* < 0.001), Affective Support (*β* = −0.419, *p* < 0.001), and contact time with patient (*β* = 0.201, *p* = 0.042) (R^2^ = 0.602).

**Conclusion:**

Caregivers of AN patients experienced heavy burdens and manifested poor mental health conditions. The severity of the patient’s symptoms from the family’s perspective and the greater use of emotion-oriented coping were associated with higher burdens. Greater use of emotion-oriented coping, less affective support and longer contact with patients were related to worse mental health conditions. Interventions to promote caregivers’ adaptive coping styles may help reduce their caregiving burden and improve their mental health.

## Background

Anorexia nervosa (AN) is one of the categories of eating disorders for which the main symptoms are a drive for thinness, fear of weight gain, extreme food restriction and severe weight loss [1]. AN is frequently accompanied by serious somatic comorbidity due to low weight and malnutrition, as well as psychiatric and social comorbidity. Without effective treatment, it often continues for many years, and the patient’s motivation for treatment often remains low [2]. Evidence of an effective treatment for AN has yet to be established, especially for adult patients [3, 4]. Families living together with AN patients are often involved in their obsessional thoughts and behaviors concerning weight, body shape and food, and deal with such symptoms and problematic behaviors for a long period of time. For this reason, family members of patients with eating disorders, particularly those who are in charge of the patient’s care (caregivers) suffer from a large mental burden. This burden is often detrimental to the caregivers’ mental health, causing depression or anxiety [5]. Furthermore, the burdens on caregivers of AN patients are even larger than those of caregivers of patients with schizophrenia [6] and bulimia nervosa [5].

The burden of caring for patients can be classified into objective burdens, such as expenses, and subjective burdens, such as mental pain or cognitions about care [7]. Most of the studies about family members of patients with eating disorders have focused on the subjective burdens of care (caregiving burden) [5]. Another important index of the burden of caregivers is their mental health conditions, which includes depression and anxiety. Previous studies have found a strong association between the caregiving burden and mental health conditions, and the influence is thought to be mutual. In other words, a high caregiving burden worsens mental health conditions [8, 9], and as mental health conditions, such as depression and anxiety, become worse, the negative aspects of care are felt more strongly [10, 11]. Thus, it is important to assess both the caregiving burden and mental health conditions in family caregivers.

In order to develop an effective intervention to reduce the family’s caregiving burden, it is necessary to identify the factors that predict the caregiving burden and mental health conditions. Previous studies [5–7, 12–18] reported factors associated with the caregiving burden. Those include demographic factors such as the caregivers’ education and marital status, the symptoms and severity of the patient’s illness, length of contact with the patient, and the patient’s refusal of the caregiver’s support. These factors also include expressed emotion (EE) which means that the caregiver is critical of the patient or emotionally entangled, family functioning, and the quantity and quality of social support for the caregiver. Furthermore, it has been indicated that use of a non-adaptive stress coping style is a unique predictor for both the caregiving burden and mental health conditions [19].

However, all previous studies were conducted in Western countries; no study has been reported on the burden of caregivers of AN patients in Japan. In addition, only limited studies have been done on the role of caregivers’ stress coping styles and social support for their burden and mental health, which could provide valuable information in developing effective interventions and support for AN families.

This study investigates the caregiving burdens and mental health conditions in family members of AN patients in Japan. It examines factors associated with the severity of burden and distress, particularly the role of coping style and social support.

## Methods

### Participants

The purpose and methods of this study were explained to 130 AN outpatients of the Institute of Women’s Health, Tokyo Women’s Medical University, between August 2012 and March 2014. The participants were the principal caregivers of 104 patients (80.0 %) who gave consent. The principal caregiver was defined as a member of the patient’s family who was providing the most care for the patient, as recognized by both the patient and the caregiver. The study was approved by the ethics committees of both the National Center of Neurology and Psychiatry and the Tokyo Women’s Medical University. Written informed consent was obtained from the participating caregivers.

### Procedure

Each caregiver was administered a set of self-report questionnaires, and the patient’s basic information was collected from the doctor in charge of the patient’s care. The set of questionnaires was distributed to the caregiver either through the patient or directly, and the response was collected by mail.

### Assessment

The caregiver was evaluated using demographic information, including the caregiver’s age, his/her relationship with the patient, working situation, educational achievement, marital status, whether the patient fundamentally refused the caregiver’s support, and length of contact time with the patient in a day. We evaluated contact time by inquiring, “How many times on average do you spend with the patient each day.” Participants choose a response from the options; less than 3 h, from 3 h to less than 6 h, more than 6 h. The patient was evaluated for age, height, weight, the lowest weight in the past at the current height, AN subtype, history of hospitalization, disease duration, and comorbid mental disorders. The patient’s BMI (Body Mass Index) was calculated based on the height and weight. In addition, the following six scales were administered to the caregiver in order to evaluate caregiving burden, psychological distress, stress coping style, social support, severity of the patient’s symptoms from the family’s perspective, and family functioning.

#### The Zarit Caregiver Burden Interview (J-ZBI_8)

The Zarit Caregiver Burden Interview assesses the caregiving burden [20]. The 8-item short Japanese version (J-ZBI_8) had been validated [21] and was used in this study. Each item is evaluated on a five-point Likert scale from 0 (Never) to 4 (Nearly always). A higher score indicates a higher caregiving burden.

#### General Health Questionnaire (GHQ-28)

The GHQ assesses psychological distress, and more specifically the presence of psycho-neurotic symptoms [22]. The 28-item Japanese version was used in this study. The scale is rated on a 4-point Likert scale, and uses two scoring methods. Using the Likert method, “not at all” = 0, “no more than usual” = 1, “rather more than usual” = 2 and “much more than usual” = 3. Using the GHQ method, “not at all” = 0, “no more than usual” = 0, “rather more than usual” = 1 and “much more than usual” = 1. The cutoff point of the GHQ method score was used for screening of a possible mental disorder and the Likert method score was used for other statistical analyses.

#### Coping Inventory for Stressful Situations Japanese Version (CISS)

The CISS measures typical coping styles in stressful situations. It consists of three subscales, named Task-Oriented Coping, Emotion-Oriented Coping, and Avoidance-Oriented Coping, each of which is composed of 16 items, making a total of 48 items [23]. The Japanese version has the same subscales, and its reliability and validity have been confirmed [24]. Each item is evaluated on a five-point Likert scale from 1 “never used” to 5 “always used”. A higher score for each subscale indicates that the corresponding coping style is used more often.

#### Social Network Questionnaire (SNQ)

The SNQ measures social support and is composed of four subscales: Social Contacts (4 items), Practical Support (3 items), Affective Support (5 items), and Partner Support (2 items). This scale was developed for a large-scale study on caregivers of patients with schizophrenia in Europe, and has been translated into multiple languages [25–27]. The scale was translated and used with permission from the author for this study. The items from the Partner Support subscale were not used for our analysis because the concept of partner differs between Japan and Europe.

#### Anorectic Behavior Observation Scale (ABOS)

The ABOS is a 30-item questionnaire that evaluates the patient’s eating disorder and cognitive problems based on the family’s actual observations [28]. The Japanese version has been validated [29]. Each item asks about the patient’s condition in the past one month and is scored as 2 if the content of the problem is certainly present, as 1 if the content has not actually been seen or its presence is not certain, and as 0 if the content is certainly not applicable. A higher score indicates that the symptoms are more severe from the family’s perspective.

#### General Functioning Subscale of the McMaster Family Assessment Device (GF-FAD)

The FAD is based on the McMaster Model of Family Functioning, and has been widely used in the area of mental illness [30] and validated in Japanese [31]. The General Functioning Subscale of the FAD (GF-FAD) consists of 12 items scored from 1 to 4. A higher score indicates that the respondent sees the family’s functioning as poorer

### Statistical analyses

In order to find factors related to the caregiving burden (J-ZBI_8) and mental health conditions (GHQ), associations with each scale score, caregiver’s age, patient’s age, age of onset, BMI, lowest BMI, and duration of illness were examined using Pearson’s correlation coefficients. Student’s t-tests or one-way ANOVAs were used to compare the mean scores of the caregiving burden and psychological distress regarding the patients’ and caregivers’ attributes. Multiple linear regression analysis (stepwise method) was conducted using factors that had significant associations (*p* < 0.05) in the analysis above as independent variables, and the ZBI and GHQ as the dependent variables. Alpha was set at 0.05. PASW Statistics 18 was used as the statistical software.

## Results

### Clinical and demographic data of patients and caregivers

Responses were obtained from 79 caregivers (response rate of 76.0 %). The clinical and demographic characteristics of the patients and their caregivers are shown in Table 1. Regarding the patients’ characteristics, all were females, the mean (± SD) age was 26.6 ± 7.9 years, the mean age of onset of AN was 17.1 ± 4.1 years, and the mean disease duration was 8.8 ± 6.1 years. Of the patients, 45 (57.0 %) had been ill for longer than 6 years and 31 (39.2 %) had been ill for longer than 10 years. Therefore, this study included subgroups of chronic patients. The mean current body mass index (BMI) was 14.6 ± 3.2 kg/m^2^, and the lowest BMI was 12.1 ± 2.0 kg/m^2^. Forty-eight patients were the restricting type (60.8 %) and 31 were the binge-eating/purging type (39.2 %). Fifty-one patients (64.6 %) had experienced hospitalization, and 20 (25.3 %) had comorbid mental, developmental, or personality disorders. The mean age of the caregivers was 56.0 ± 8.0 years. Five (6.3 %) were the patients’ fathers, 70 (88.6 %) were mothers, three (3.8 %) were spouses, and one (1.3 %) was a grandmother. Seventy-two caregivers (91.1 %) live together with their family patients. Regarding contact time with the patient in a day, 14 (17.7 %) reported less than 3 h, 36 (45.6 %) reported between 3 and 6 h, and 29 (36.7 %) reported 6 h or more. Six respondents (7.6 %) stated that the patient fundamentally rejected the caregiver’s support.

**Table 1:** Clinical and demographic data of patients and their caregivers

Subjects		n	%	Mean	SD
Patient variables					
Age (y)				26.6	7.9
Age of onset (y)				17.1	4.1
Current BMI (kg/m^2^)				14.6	3.2
Lowest BMI (kg/m^2^)				12.1	2.0
Disease duration (y)				8.8	6.1
AN subtype	AN-R	48	60.8		
	AN-BP	31	39.2		
Hospitalized	Yes	51	64.6		
	No	28	35.4		
Comorbidities	Yes	20	25.3		
	No	59	74.7		
Caregiver variables					
Age (y)				56.0	8.0
Relationship	Father	5	6.3		
	Mother	70	88.6		
	Other	4	5.1		
Working situation	Full-time	18	22.8		
	Part-time	22	27.8		
	Unemployed	39	49.4		
Educational level	High school	26	32.9		
	Junior college Technical school	35	44.3		
	College	18	22.8		
	University				
Living together with patient	Yes	72	91.1		
	No	7	8.9		
Contact time with patient in a day	<3 h	14	17.7		
	3 -6 h	36	45.6		
	>6 h	29	36.7		

### Scores for the assessment scales

The scores for the scales administered are displayed in Table 2. The total score for the caregiving burden (J-ZBI_8) was 12.4 ± 7.0 (mean ± SD). The mean total score for psychological distress (GHQ) was 31.6 ± 13.7 using the Likert scoring method of 0–3; and 9.2 ± 7.0 using the GHQ scoring method of 0–1. Forty-eight respondents (60.7 %) exceeded the cutoff point (6/7) indicating a high risk of mental disorders. Regarding stress coping styles (CISS), the mean score for Task-Oriented Coping was 52.6 ± 6.8, Emotion-Oriented Coping was 51.9 ± 4.6, and Avoidance-Oriented Coping was 40.9 ± 9.5. In terms of social support (SNQ), the mean score for Social Contact was 9.1 ± 2.3, Practical Support was 8.8 ± 2.6 and Affective Support was 14.9 ± 3 .0. Regarding the evaluation of eating disorder symptoms by the family (ABOS), the mean score was 22.2 ± 10.4. The mean total score for family functioning (GF-FAD) was 25.6 ± 6.3.

**Table 2:** Scale scores for caregivers of patients with anorexia nervosa

Scales	*n*	Mean	SD	Min.	Max.	Range of scores
J-ZBI_8	79	12.4	7.0	0	27	0-32
GHQ(Likert method)	79	31.6	13.7	4	70	0-84
GHQ(GHQ method)	79	9.2	7.0	0	28	0-28
CISS-Task	75	52.6	6.8	35	73	16-80
CISS-Emotion	79	51.9	4.6	48	69	16-80
CISS-Avoidance	78	40.9	9.5	20	63	16-80
SNQ-Social contacts	79	9.1	2.3	4	13	4-16
SNQ-Practical support	54	8.8	2.6	2	12	3-12
SNQ-Affective support	69	14.9	3.0	7	20	5-20
ABOS	77	22.2	10.4	4	48	2-60
GF-FAD	79	25.7	6.3	12	44	12-48

*J-ZBI_8* Zarit caregiver burden interview, *GHQ* general health questionnaire, *CISS* coping inventory for stressful situations. *CISS-Task* task-oriented coping of CISS, *CISS-Emotion* emotion-oriented coping of CISS, *CISS-Avoidance* avoidance-oriented coping of CISS, *SNQ* social network questionnaire, *ABOS* anorectic behavior observation scale, *GF-FAD* general functioning subscale of the mcmaster family assessment device

### Relationship of patient and caregiver characteristics with the caregiving burden and caregiver’s mental health conditions

The association between the patients’ and caregivers’ attributes and caregiving burden was investigated. A significant difference in J-ZBI_8 scores was found between the groups based on contact time with the patient (F (2, 76) = 3.19, *p* = 0.047). A post-hoc multiple comparison using Dunnett’s test (5 % level of significance) revealed that the ZBI total score was significantly higher for the 6 h or more contact group than for the under 3 h contact group. A relationship of the total GHQ score (Likert method) with the length of time in contact with the patient was also found (F (2, 76) = 9.39, *p* < 0.001). The 6 h or more contact group had a higher total GHQ score than the other two groups. No significant relationship was found between the other attributes and the caregiving burden or psychological distress conditions.

### Factors that predict caregiving burden and mental health conditions

The correlations between J-ZBI_8, total GHQ and other scale scores are displayed in Table 3. Furthermore, the correlations between the four subscale scores of the GHQ, Somatic Symptoms, Anxiety and Insomnia, Social Dysfunction and Severe Depression and other scale scores are displayed in Table 4. There was a relatively high positive correlation between J-ZBI_8 and the total GHQ score (r = 0.60, *p* < 0.0001).

**Table 3:** Pearson’s correlation coefficients between scales

	1	2	3	4	5	6	7	8	9
1. J-ZBI_8									
2. GHQ	.61***								
3. CISS-Task	-.19	-.14							
4. CISS-Emotion	.44**	.55***	-.08						
5. CISS-Avoidance	.05	.04	.31**	.03					
6. SNQ-Social contacts	-.35**	-.33**	.13	-.03	.26				
7. SNQ-Practical support	-.33*	-.24	.33*	-.01	.22	.42**			
8. SNQ-Affective support	-.33**	-.48***	.31*	-.15	.34**	.58***	.50***		
9. ABOS	.50**	.41**	-.09	.26*	.03	-.41***	-.30*	-.42***	
10. GF-FAD	.23*	.25*	-.10	.13	-.02	-.21	-.37***	- .31**	.22

*J-ZBI_8* Zarit caregiver burden interview, *GHQ* general health questionnaire, *CISS* coping inventory for stressful situations, *CISS-Task* task-oriented coping of CISS, *CISS-Emotion* emotion-oriented coping of CISS, *CISS-Avoidance* avoidance-oriented coping of CISS, *SNQ* social network questionnaire, *ABOS* anorectic behavior observation scale, *GF-FAD* general functioning subscale of the mcmaster family assessment device

*p < 0.05, **p < 0.01, ***p < 0.001

**Table 4:** Correlations between subscales of the GHQ and other scales

	Total	Somatic symptoms	Anxiety/insomnia	Social dysfunction	Severe depression
J-ZBI_8	.61***	.38**	.56***	.56***	.55***
CISS-Task	-.14	-.02	-.09	-.25*	-.14
CISS-Emotion	.55***	.28*	.59***	.30**	.56***
CISS-Avoidance	.04	.07	.18	-.25*	.02
SNQ-Social contacts	-.33**	-.31**	−0.28*	-.25*	-.25*
SNQ-Practical support	-.24	-.15	-.17	-.29*	-.23
SNQ-Affective support	-.48***	-.41***	-.33**	-.41***	-.37**
ABOS	.41***	.43***	.39***	.12	.31**
GF-FAD	.25*	.21	.19	.25	.19

*GHQ* general health questionnaire, *J-ZBI_8* Zarit caregiver burden interview, *CISS* coping inventory for stressful situations, *CISS-Task* task-oriented coping of CISS, *CISS-Emotion* emotion-oriented coping of CISS, *CISS-Avoidance* avoidance-oriented coping of CISS, *SNQ* social network questionnaire, *ABOS* anorectic behavior observation scale, *GF-FAD* general functioning subscale of the mcmaster family assessment device

*p < 0.05, **p < 0.01, ***p < 0.001

The factors with a significant correlation with J-ZBI_8 were Emotion-Oriented Coping from the CISS; Social Contacts, Practical Support and Affective Support from the SNQ; ABOS; and GF-FAD. Stepwise multiple linear regression analysis was conducted applying these factors and “contact time with patient” as the independent variables and the total score of the J-ZBI_8 as dependent variables. The factors that significantly predicted the J-ZBI_8 were ABOS (*β* = 0.47, *p* < 0.001) and Emotion-Oriented Coping (*β* = 0.38, *p* = 0.002) (Table 5). These two factors predicted 40.1 % of the variance of the caregiving burden.

**Table 5:** Multivariate regression analyses of variables predicting ZBI total score (step-wise method)

	B	SE	β	t	P	R^2^
Model 1						
ABOS	.352	.087	.510	4.021	<0.001	.260
Model 2						
ABOS	.325	.080	.472	4.064	<0.001	.401
CISS-Emotion	.212	.065	.377	3.247	.002	

*J-ZBI_8* Zarit caregiver burden interview, *ABOS* anorectic behavior observation scale, *CISS-Emotion* emotion-oriented coping of coping inventory for stressful situations

Similarly, the factors that predicted the total GHQ score were: Emotion-Oriented Coping, Social Contact, Affective Support, ABOS, and GF-FAD. Stepwise multiple linear regression analysis was performed with these factors and contact time with patient as the independent variables and psychological distress as the dependent variable. The results showed that Emotion-Oriented Coping (p < 0.001), Affective Support (*p* < 0.001), and contact time with patient (*p* = 0.042) significantly predicted the total GHQ score (Table 6). These three factors predicted 60.2 % of the variance of the total GHQ.

**Table 6:** Multivariate regression analyses of variables predicting GHQ total score (step-wise method)

	B	SE	β	t	P	R^2^
Model 1						
CISS-Emotion	.695	.126	.630	5.509	<0.001	.398
Model 2						
CISS-Emotion	.606	.111	.550	5.469	<0.001	.563
SNQ-Affective support	−1.772	.430	-.414	−4.122	<0.001	
Model 3						
CISS-Emotion	.575	.108	.522	5.331	<0.001	.602
SNQ-Affective support	−1.791	.415	-.419	−4.321	<0.001	
Contact time with patient	2.330	1.110	.201	2.098	.042	

*GHQ* general health questionnaire, *CISS-Emotion* emotion-oriented coping of coping inventory for stressful situations, *SNQ-Affective support* affective support of social network questionnaire

## Discussion

The results of this study show that the majority of caregivers for AN patients suffered a heavy burden of care and tended to have poor mental health. Furthermore, the factors that predicted a high caregiving burden were severity of eating disorder symptoms from the family’s perspective, and use of emotion-oriented stress coping strategies. Likewise, factors which predicted poor mental health conditions were use of emotion-oriented stress coping strategies, lack of affective support, and longer contact time with the patient.

The principal caregiver in this study was defined as the person who takes care of the patient most among the family. The vast majority of the principal caregivers were the mothers of the patients (88.6 %), indicating that mothers are ordinarily bearing the care burden of AN patients. The mean total score of the J-ZBI_8 (12.4 ± 7.0) for the principal caregivers of AN patients was higher than the mean score (9.3 ± 7.2) for the principal caregivers who answered that they were “troubled by care” of elderly patients requiring at-home nursing care [21], and comparable to the mean score of the J-ZBI_8 (13.3 ± 7.8) for mothers who live with schizophrenic patients in Japan [32].

In addition, 60.8 % of respondents exceeded the cutoff point for GHQ scores indicating a high rate of mental health problems among the principal caregivers. The mean GHQ Likert score (31.6 ± 13.7) for the principal caregivers in our Japanese study was comparable to that for caregivers of eating disorder patients (27.7 ± 6.2) and even higher than that for schizophrenic patients (16.4 ± 8.0) reported in a study in England [3]. Thus, caregiving for AN patients posed a significant risk for mental health problems. Our results support the previous findings that caregivers of eating disorder patients had high levels of depression, high levels of anxiety, and impaired mental health [5, 18].

The only demographic factor which correlated with the level of caregiving burden as well as mental health conditions was the length of contact time with the patient. One previous study found a significant correlation between the length of contact and caregiving burden [17], while others did not [19, 32]. Our results showed that longer contact time with a patient predicts poorer mental health in the caregiver. This may be related to Japanese circumstances surrounding patients with eating disorders and their families. At present, both in-patient and outpatient treatment facilities for eating disorders are quite insufficient [33] and day-patient treatments are rarely available for eating disorders. Therefore, it is not rare that patients stay home almost all day and their families have to take care of the patients for long hours that may lead to their mental exhaustion. We assessed the contact time as the average time caregivers spent with the patient par day. As a result, we did not assess if contact time signified the time that caregivers spend in actually caring for patients, or the time caregivers and patients were in the same room. Therefore, further studies are necessary to clarify the influence of the nature of contact time on caregiver’s burden.

The severity of the patients’ symptoms as evaluated by their families, measured by the ABOS, was the strongest predictor of the caregiving burden. Symptom severity has been reported repetitively to be an important predictor of the subjective burden of care not only for patients with eating disorders, but also for other mental illness such as bipolar disorders, dementia, schizophrenia and spectrum disorders [34–37]. On the other hand, our study did not find any association between caregiving burden and the AN subtype, BMI, comorbid mental disorders and patient diagnostic information as evaluated by the attending physician. The ABOS includes items about behaviors that can be observed only by the family, such as getting angry or leaving the table during a meal, or dislike of visitors because of feeling an obligation to eat. These embarrassing actions may strongly affect family life and make the family feel burdened. Our findings support the view that the observation of symptoms by the family is indispensable in the assessment of the family burden [18, 29].

Emotion-oriented stress coping was one of the predictors of caregiving burden independent of the patient’s symptoms. It also was the strongest predictor of mental health conditions. A limited number of studies have investigated the relationships between stress coping styles and care burdens among caregivers of eating disorder patients. Coomber et al. [19] found that non-adaptive stress coping predicted both the caregiving burden and mental health conditions. They used the Brief COPE [38] as a scale of stress coping, and categorized coping styles into non-adaptive and adaptive coping. On the other hand, the CISS used in our study examines stress coping using three subscales: Task-Oriented Coping, Emotion-Oriented Coping, and Avoidance-Oriented Coping. Stress coping that focuses on emotions generally includes both adaptive coping such as positive interpretations of the situation, and non-adaptive coping such as negative emotional expressions involving self-blame and venting [39]. However, the emotion-oriented coping documented by the CISS is considered to be a non-adaptive aspect of coping. Emotion-oriented coping on the CISS has been related to high care burdens on family members of Alzheimer’s patients [40], and was indicated to be a factor of professionals’ burnout [41]. Our study found that emotion-oriented non-adaptive stress coping has a negative effect on both the caregiving burden and the mental health of caregivers of AN patients as well.

In addition, qualitative studies have demonstrated that caregivers of eating disorder patients are more likely to blame themselves, criticize their patients and feel despair [40, 42]. The Emotion-Oriented Coping items of the CISS include self-criticism, criticism of others, and coping that is swayed by mood. Our study suggests that these aspects of stress coping increase the care burden.

On the other hand, neither task-oriented nor avoidance-oriented coping correlated with the caregiving burden and mental health conditions. However, task-oriented coping had a positive correlation with practical support and affective support, while avoidance-oriented coping had a positive correlation with affective support. This might be interpreted as indicating that use of task-oriented coping strategies could lead to seeking practical and emotional support from others, and that avoidance-oriented coping strategies, such as diversions or social interactions, lead to receiving affective support from others.

The caregiving burden correlated negatively with social contacts, affective support, and practical support. Mental health conditions showed negative associations with social contacts and affective support. Past studies have also indicated that obtaining social support is protective against the caregiving burden and mental health impairment [19, 25, 27, 32]. The multiple regression analysis in our study indicated that, among the forms of social support, affective support was a particularly important predictor of mental health conditions. It is possibly helpful for caregivers to share problems and emotions with others and to have trustworthy friends and relatives as part of the process of maintaining their mental health.

The analysis of the correlations of the GHQ28 subscales with other scales yielded results generally similar to those for the GHQ28 total score. This is likely because the GHQ28 subscales scores and total score show strong correlations with each other. However, social dysfunction showed negative correlations with task-oriented and avoidance-oriented coping and practical support, while the total score did not. In contrast, social dysfunction did not correlate with the ABOS while the total score did. This can be interpreted as an interactive effect. In another words, higher social dysfunction may disturb stress coping, while sparse interpersonal interactions may make it difficult to obtain practical support. Conversely, poor stress copings and insufficient practical support may enhance social dysfunction. Thus, the social dysfunction of a caregiver is strongly related to his/her stress coping and social support rather than to the patient’s condition.

There are several limitations to our study. First, the present results should be generalized only with caution to other Japanese families of AN patients because the study participants were exclusively family members of patients receiving outpatient treatment at a university hospital in a metropolitan area. When interpreting the result of this study, special consideration should be given to the fact that the sample of this study consisted of a subgroup of severe and enduring patients with a relatively long mean illness duration, as well as a low current and minimal BMI. In addition, the sample size in this study is modest. It is necessary to conduct a study with a larger sample size with an appropriate sampling method in order to obtain more generalizable findings. Second, it is difficult to compare our current results with former studies, since we used scales for caregiving burden, stress coping and social support that are different from those used in previous studies. Third, our cross-sectional design does not indicate a causal relationship of the caregiving burden and mental health conditions with possible predictive factors.

## Conclusion

Family members who provide principal care to AN patients quite often suffer from heavy caregiving burdens and poor mental health. The factors that predict a high caregiving burden include severity of eating disorder symptoms from the family’s perspective and more use of emotion-oriented stress coping strategies. The factors that predict poor mental health conditions of caregivers are more use of emotion-oriented stress coping, less affective support from one’s surroundings, and longer contact time with the patient. Our results suggest that interventions to promote caregivers’ adaptive coping styles may reduce their caregiving burden and improve their mental health.
